# Gerstmann’s Syndrome and Limb Apraxia: A Single Case Study

**DOI:** 10.1093/arclin/acaf083

**Published:** 2025-09-18

**Authors:** Sara Bertagnoli, Maddalena Beccherle, Riccardo Danese, Cristina Bulgarelli, Valeria Gobbetto, Greta Vianello, Elena Rossato, Valentina Moro

**Affiliations:** Social and Cognitive Neuroscience Laboratory, Department of Psychology, University La Sapienza, Roma, Italy; NPSY-Lab.VR, Department of Human Sciences, University of Verona, Verona, Italy; NPSY-Lab.VR, Department of Human Sciences, University of Verona, Verona, Italy; Physical Medicine and Rehabilitation Unit, IRCSS Sacro Cuore Don Calabria, Verona, Italy; Physical Medicine and Rehabilitation Unit, IRCSS Sacro Cuore Don Calabria, Verona, Italy; NPSY-Lab.VR, Department of Human Sciences, University of Verona, Verona, Italy; Physical Medicine and Rehabilitation Unit, IRCSS Sacro Cuore Don Calabria, Verona, Italy; NPSY-Lab.VR, Department of Human Sciences, University of Verona, Verona, Italy; Physical Medicine and Rehabilitation Unit, IRCSS Sacro Cuore Don Calabria, Verona, Italy

**Keywords:** Left–right confusion, Finger agnosia, Acalculia, Agraphia, Apraxia, Body representation

## Abstract

**Objective:**

Gerstmann’s syndrome (GS) represents a still debated clinical condition, in terms of both symptoms’ evolution and neural correlates. In depth, repeated neuropsychological assessments along with advanced methods of lesion analysis can contribute to a better understanding of the syndrome and clinical diagnosis.

**Method:**

The study reports a patient suffering from GS and limb apraxia following a left hemisphere lesion. Two in-depth assessments, at two and four months from the lesion onset, in addition to video material, document the symptoms over time. An in-depth analysis of the grey and white matter lesions was carried out with 3D reconstruction and a disconnection map.

**Results:**

The patient shows the characteristic tetrad of GS symptoms in both subacute and chronic phases, in the absence of other clinically relevant sensorimotor or cognitive deficits. Limb apraxia persists over time as well. The neuroanatomical investigation shows the involvement of cortical damage in the inferior parietal cortex that extends to the superior parietal cortex, anteriorly to the peri-rolandic area and medially to the precuneus. Furthermore, disconnections in the fronto-parietal networks and the corpus callosum were identified.

**Conclusions:**

This single case study supports previous neuropsychological evidence and neuroanatomical findings on healthy participants, suggesting a core neural network underlying the four GS symptoms, which involves the left superior parietal lobe, the intraparietal cortices and the white matter parietal and fronto-parietal tracts. Furthermore, the involvement of the three branches of the superior longitudinal fasciculus explains the co-occurrence of limb apraxia.

## INTRODUCTION

The nature of Gerstmann’s syndrome (GS, Gerstmann, 1924, 1940), also known as the “angular gyrus syndrome” or “Gerstmann-Badal syndrome” (Triarhou, 2008), is debated in neuropsychology, both in terms of its clinical characterizations and its neuroanatomical correlates.

In the original report, the syndrome was described as the association of four symptoms: left–right disorientation (i.e., difficulties in localizing left and right in symmetrical body parts), finger agnosia (i.e., disorders in recognizing, naming and indicating the individual’s own and other people’s fingers), dyscalculia (i.e., deficits in arithmetical operations with even simple numbers), and dysgraphia (i.e., difficulties in accessing the graphemic representation of letters). However, GS often emerges in an incomplete form (i.e., with three of the four symptoms, Mayer et al., 1999) or in association with other cognitive deficits, such as aphasia, anomia, constructional apraxia, hemianopia, and autotopagnosia (see Rusconi, 2018). For this reason, and considering its rarity, the hypothesis has been advanced that the simultaneous occurrence of the tetrad of symptoms does not represent a unique syndrome but is either simply coincidental or due to the anatomical proximity of the different networks mediating these four functions (Basagni et al., 2021; Wingard et al., 2002). Therefore, a general framework to systematically assess GS would be useful for clinicians and researchers to better understand the symptom co-occurrence and identify disorders that nowadays often remain undiagnosed (Ardila, 2020).

Another matter of debate regards the neuroanatomical correlates of GS. Gerstmann suggested the left angular gyrus as the node for the combination of symptoms (Gerstmann, 1940). However, GS has also been reported following right hemisphere lesions (Hayashi et al., 2013; Moro et al., 2009; Moore et al., 1991; Sauguet et al., 1971) and other left-hemisphere lesioned areas, such as the inferior frontal gyrus (João et al., 2017; Tanabe et al., 2020) or the thalamus (Casado et al., 1995). More recently, the possibility that GS is the result of subcortical fronto-parietal and intraparietal disconnections rather than direct damage to the parietal lobe has been discussed (Al-Samaraie, 2024; Kleinschmidt & Rusconi, 2011; Ranzini et al., 2023; Rusconi et al., 2009).

This last possibility would explain the differences in clinical manifestations, which could arise from lesions involving different parts of the network. This interpretation of the syndrome has not been easy to investigate to date, mainly due to the rarity of the syndrome. Literature on GS refers mainly to single case studies, with wide variability in clinical symptoms and lesions (Ardila, 2020; Tekgol Uzuner et al., 2020).

Furthermore, only recently, investigations into brain lesions have overcome the limitations of the standard methodology used in previous studies, allowing researchers to analyze the contribution of both grey and white matter lesions in the pathogenesis of neuropsychological syndromes. By means of direct and indirect investigations of white matter, it is now possible to go beyond the role of discrete, direct cortical lesions in the patients’ symptoms and identify the contribution of structures that are apparently spared but are somehow involved via white matter disconnections (Beccherle et al., 2023; Bertagnoli et al., 2022; Pacella et al., 2020; Thiebaut de Schotten et al., 2015).

This single case study describes a patient presenting all four symptoms of GS associated with limb apraxia with the aim of analyzing the symptoms’ evolution over time and offering a framework for a systematic assessment of the syndrome. Along with in-depth neuropsychological assessments, carried out at two and four months from the lesion onset, an advanced lesion analysis investigation was performed to investigate white matter disconnections indirectly and test the hypothesis of a diffuse neural network underlying GS.

## MATERIALS AND METHODS

### Case Report

CL is a 62-year-old, right-handed man (13 years of education), who sought medical assistance following a left hemisphere stroke, with consequent right motor hemiparesis and somatosensory deficits. Neuroimaging (CT and MRI) revealed a parenchymal hematoma in the parietal area, herniating in the left medial temporal area. This spontaneously resolved in the following weeks, leading to a progressive improvement of the patient’s clinical conditions. Six weeks after the lesion onset, he was moved to a rehabilitation unit.

He showed left upper and lower limb hyposthenia (movement against gravity, but not against resistance) and somatosensory deficits; he was verbally fluent, but sometimes his speech seemed confused and unclear. Signs of right personal neglect and limb apraxia were reported in the clinical report. He complained that he could not control his right hand, which did not respond to his will.

When we met CL two months after the lesion onset, his motricity had largely recovered. He could move his upper and lower right limbs against resistance, although a reduction in strength persisted. Minimal signs of sensory deficits in the discrimination of temperature and pressure were present, along with wrist, hand and ankle proprioceptive deficits (i.e., he perceived the movements but failed to identify the direction). No signs of tactile anesthesia were recorded. Executive functions (FAB, TMT, and ACE-R attention sub-score in Table 1), language (AAT comprehension, denomination, repetition subscales, Luzzatti et al., 1987; ACE-R language subscale, Siciliano et al., 2016), and other cognitive functions (i.e., attention, temporal orientation, and visuo-spatial functions [ACE-R, Siciliano et al., 2016]) were spared. Despite the borderline performances in the ACE-R memory subscale and in the Digit Span Forward, a more in-depth assessment of memory in CL excluded specific verbal and visual memory impairments (Table 1).

**Table 1:** Results from the assessment of sensory-motor deficits and cognitive functions. CL’s scores are reported and compared to the single task’s cut-off, maximum scores(), Equivalent Scores or T-Scores

**Task**	**CL’s Scores 2 months**		**Cut-off/ES/TS (max score)**
A. Motricity index (Collin & Wade, 1990)	Right	Left	
Upper limb	**77**	100	(100)
Lower limb	**76**	100	(100)
B. Somato-sensory functions	Right	Left	
Nottingham sensory assessment (Lincoln et al., 1998)			
Light touch	18	18	(18)
Temperature	**16**	18	(18)
Pinprick	18	18	(18)
Pressure	**16**	18	(18)
Tactile localization	18	18	(18)
Bilateral simultaneous touch	**14**	18	(18)
Proprioception	**13**	21	(21)
C. Cognitive functions			
General cognition (screening)			
Mini mental state examination (Measso et al., 1993)	28.49		23.8
ACE-R (Siciliano et al., 2016)	81.77		71.78
Attention and orientation	16.91		ES 2
Memory	14.63		ES 1
Verbal fluency	9.3		ES 2
Language	16.91		ES 4
Visuo-spatial functions	14.23		ES 3
Attention and executive functions			
Trail making test (Giovagnoli et al., 1996)			
TMT A	57		ES 2
TMT B	154		ES 2
TMT B-A	97		ES 2
Frontal assessment battery (Appollonio et al., 2005)	14.5		13.4
Digit span forward (Monaco et al., 2013)	**3.92**		**ES 0**
Digit span backward (Monaco et al., 2013)	3.87		ES 3
Language			
AAT (Luzzatti et al., 1987)			
Token test (errors)	2		TS 73 [9]
Oral comprehension	118		TS 73 [9]
Denomination	118		TS 77 [9]
Repetition	144		TS 64 [8]
Memory			
Story recall (Carlesimo et al., 2002)			
Immediate	6.5		3.1
Delayed	3.3		2.39
Corsi spatial span test (Orsini et al., 1987)	3.75		3.5
Corsi spatial supraspan test (Spinnler & Tognoni, 1987)	20.09		5.75
Spatial neglect			
BIT – conventional subtests (Wilson et al., 1987)	144		129
Personal neglect			
Fluff test (Cocchini & Beschin, 2022)	15		±13.3
Comb & razor (compact) test (McIntosh et al., 2000)			
Comb (right bias)	**0.19**		>0.11
Razor (right bias)	**0.37**		>0.11
Anosognosia for hemiplegia			
MUNA (Moro et al., 2021)–Total score	2		≥27
Explicit awareness	0		≥11
Implicit awareness	0		≥0.75
Sense of ownership	0		≥3
Sense of agency	1		≥2
Emotional reactions to paralysis	1		≥6
Robust score	0		≥13

*Note:* The pathological scores are reported in bold. ES = Equivalent Score (Capitani & Laiacona, 1988, 1997; Facchin et al., 2022; ES 0 = pathological; ES 1 = uncertain result (further investigation is necessary); ES ≥ 2 normal range); TS = T-Score (in squared brackets the ES of AAT, max score: 10); ACE-R = Addenbrooke’s Cognitive Examination -Revised; AAT = Aachen Aphasia Test; BIT = Behavioral Inattention Test; MUNA = Motor Unawareness Assessment.

A bias toward the left side emerged from the Comb and Razor test (McIntosh et al., 2000) without any other signs of spatial or personal neglect. The patient was aware of his sensorimotor deficits and did not show disorders relating to agency or contralesional limb ownership. His emotional responses appeared congruent with his concerns associated with his condition. He made mistakes during daily life activities (e.g., during meals or activities related to personal hygiene and grooming) and showed signs of confusion between his left and right hands when asked to perform a specific action. An evaluation of these symptoms was administered (see the following sections).

### Gerstmann’s Syndrome Assessment

An assessment of the patient’s symptoms regarding left–right disorientation, finger agnosia, acalculia and dysgraphia was repeated twice, at two and four months from the lesion onset (see Table 2 and Supplementary material online, Video 1 – SM 1).

#### Left–right disorientation

Benton’s test was used to assess the patient’s difficulties with left–right orientation in relation to his body (self; 12 items, 6 on each side of the body), another person’s body (other; 8 items) and a drawing of a model facing the patient (figure; 8 items). CL was verbally asked to point to left or right body parts (e.g., eye, knee, not fingers or toes). For half of the items, the patient was free to use either hand (i.e., one stage command), whereas for the others, he was asked to use a specific hand for a specific body part (i.e., two stage command). One point was given for each correct identification.

#### Finger agnosia

In the Benton’s Finger localization battery (1959; 2000), individual fingers are stimulated by the examiner in a predetermined random order, and the patient is asked to denominate the finger touched. Ten trials for each hand are carried out in two different conditions: (a) with the aid of vision (self-visible hand) and (b) without the aid of vision (self-hidden hand). Furthermore, a third condition was added (other condition) in which the examiner touched the finger of another person next to the patient (i.e., a not mirrored perspective). Finally, a task involving identification without denomination (i.e., digital identification) was carried out, requiring the patient to lift the finger being touched by the examiner (not visible to the patient). A task involving intermanual transmission was also administered (Moro et al., 2015; Pacella et al., 2021).

#### Dyscalculia and dysgraphia

The calculation subtest of the ACE-R (Siciliano et al., 2016) and simple two-digit additions and subtractions were executed. Furthermore, the WAIS subtest for Arithmetical Reasoning was administered (Orsini & Pezzuti, 2013). *Dysgraphia* was clinically investigated.

### Apraxia Assessment

The following tests were administered to assess apraxia.

#### Ideomotor apraxia

In the Movement Imitation Test (De Renzi et al., 1980), the examiner sits in front of the patient and shows a series of 24 movements. The patient is requested to reproduce and mirror each gesture. Three dimensions differentiating the gestures are considered: (a) the limb part involved in the action (i.e., 12 hand and 12 finger movements); (b) the ability to hold a position (12 movements) or carry out a motor sequence (12 movements); and (c) the symbolic or non-symbolic nature of the gesture (i.e., 12 meaningful and 12 meaningless). Three repetitions are allowed for each gesture, with a score = 3 if the gesture is performed correctly at the first trial, 2 when it is correct at the second attempt, 1 if it is correct at the third attempt, and 0 if all the three trials are unsatisfactory (Table 3).

#### Ideational apraxia

In the Demonstration-of-Use test (*Pantomime*, De Renzi et al., 1980), the patient is requested to demonstrate the use of 10 common objects presented one at a time on the table, without touching the object. A flawless performance scores 2, a partially incorrect performance scores 1 and an incorrect execution scores 0.

#### Constructional apraxia

Three familiar figures (star, cube, and house) are shown (Carlesimo et al., 1996)*.* In the first part of the task (i.e., with planning elements), patients are asked to copy the figures freehand. The score ranges from 4 (a perfect copy) to 0 points (an unrecognizable copy). In the second part (i.e., without planning elements), to assist the patient, some graphic lines of the figure are already present on the paper on which the copy must be drawn.

#### Bucco-facial

The Bucco-facial apraxia test was used (De Renzi and Spinnler, 1966; Spinnler & Tognoni, 1987). The patient sits in front of the examiner and is asked to make 10 bucco-facial movements. Two repetitions are allowed for each trial (30 s each). A score of 2 is attributed if the movement is correctly reproduced at the first attempt, 1 if it is correctly performed at the second, and 0 in case of failure.

### Neuroanatomical Investigation

The patient’s lesion (MRI recorded at four months from lesion onset) was manually drawn on the native 3D-T1-MRI in the axial slices and checked in double-blind by two anatomists. All the slices available were drawn, and the lesion was reconstructed in the 3D region of interest (ROI) with MRIcron (https://www.nitrc.org/projects/mricron, Rorden & Brett, 2000). The 3D-T1-MRI were registered to the MNI152 template using affine and diffeomorphic deformations (Avants et al., 2011; Klein et al., 2009) by means of the “Normalization” tool (part of the BCBToolkit software; Foulon et al., 2018). To identify the grey matter structures encompassed by each lesion, the patient’s normalized lesion was compared with the AAL brain atlas. The Disconnectome Map tool (part of the BCBToolkit software; Foulon et al., 2018) was used to identify a probability of disconnection from 0% to 100% for the patient’s lesion (Thiebaut de Schotten et al., 2015). The patient’s disconnectome map was then thresholded via the fslmaths tool of FSL (https://fsl.fmrib.ox.ac.uk/fsl/fslwiki), to consider voxels with above 50% probability of disconnection.

## RESULTS

The GS and limb apraxia symptoms are shown in two videos (see Supplementary material online, SM 1 and SM 2) as well as in Tables 2 and 3.

**Table 2:** Assessment of Gerstmann’s syndrome

	CL’s Scores				Cut-off (max score)
	2 months		4 months		
A. Left–right disorientation					
Self (*n* = 12)	**9**		11		≤ 10^a^
Other (*n* = 8)	**3**		**4**		≤ 5^a^
Figure (*n* = 8)	**4**		8		(8)
B. Finger agnosia	LH	RH	LH	RH	
Digital localization (denomination)					
Self-visible hand (*n* = 10 RH; 10 LH)	10	**9**	10	10	10^b^
Self-hidden hand (*n* = 10 RH; 10 LH)	10	**6**	10	**5**	8^b^
Other/anatomical–visible hand (*n* = 10 RH; 10 LH)	10	**6**	/	/	(10)
Digital identification (*n* = 10 RH; 10 LH)	9	**7**	9	**7**	(10)
Intermanual transmission	**LH > RH**	**RH > LH**	**LH > RH**	**RH > LH**	
	9	7	10	8	(10)
C. Acalculia					
Arithmetical reasoning–Wais	**9**		**+**		(22)
Calculation test (subtest ACE-R)	**2**		**+**		(5)
D. Agraphia	**+**		**+**		

*Note:* In bold are the pathological scores. LH: left hand; RH: right hand; +: presence of deficit; >: versus (e.g., Intermanual transmission from left vs. right hand); /: information not available.

^a^For cut-off, see Ferracuti et al., 2000.

^b^For cut-off, see Gainotti et al., 1972; Gainotti & Tiacci, 1973.

**Table 3:** CL’s performance in the tests for ideomotor, ideative, and constructional apraxia

	CL’s Scores				Cut-off (max score)
	2 Months		4 Months		
	Right	Left	Right	Left	
Gesture imitation test (De Renzi et al., 1980, 1986)					
Finger gestures	**8**	**23**	**25**	30	28
Hand gestures	**13**	**22**	**22**	**24**	32
Maintaining a position	**10**	**30**	**29**	**24**	32
Motor sequence	**11**	**15**	**18**	30	27
Meaningful gestures	**16**	**27**	**27**	32	30
Meaningless gestures	**5**	**18**	**19**	**22**	29
Total score	**21**	**45**	**47**	54	53
Pantomime use of objects (De Renzi et al., 1980)	**4**	/	**16**	/	18
Copying of geometric drawings (Caltagirone et al., 1995)					
Without programming elements	**10**	/	/	/	(12)
With programming elements	70	/	/	/	(70)
Bucco-facial apraxia (De Renzi et al., 1966)	19	/	/	/	17.4
FIM (Keith et al., 1987; UDSfMR, 1993)	**30**		99		126

*Note*: In bold, the pathological scores. See the text for details. FIM = Functional Independence Measure.

### Gerstmann’s Syndrome

#### Left–right disorientation

At the first assessment, CL could not execute the task. When asked to point at a body part with one of his hands, he seemed hesitant and looked at his hands with uncertainty, without being able to choose which to use. Furthermore, when he was free to use either hand, he still confused the side of the body part. At the 4th-month evaluation, there was a limited recovery in the self and figure conditions, but left–right disorientation persisted.

#### Finger agnosia

Unilateral right finger agnosia emerged in all the tasks administered (i.e., self-visible hand, self-hidden hand and other condition). Although the performance was better when the hand was visible to the patient, at the first assessment, his score was below the cut-off, whereas a recovery was evident at the 4th-month assessment. When the patient had to identify fingers on another person’s hand, he failed the task. Note that no significant deficits in tactile perception or language disorders (ACE-R, AAT, Table 1) could explain finger agnosia. Difficulties in command comprehension were also excluded by the perfect performances referred to the left hand.

#### Dyscalculia and dysgraphia

During the execution of simple additions and subtractions, CL was slow and uncertain and also made errors. Double-digit subtractions were impaired and the patient himself complained about his difficulties (e.g., 86–7 = 89; 89–7 = 83; “Laura is 35 years old. Robert is 18. How much older is Laura than Roberto?” First response: 7 years; Second response: 25 years). Multiplication was impaired as well (“In a pack of chewing gum, there are 25 gums. How many gums are there in 8 packs?” Response: 160).

Writing was totally impaired with both left and right hands. CL needed to think about the shape of each letter. Part of the rehabilitation training focused on the recovery of his signature. Despite a partial recovery, his writing remained sloppy and slow with hesitations. As shown in Table 1, reading was spared.

### Apraxia

#### Ideomotor apraxia

At the first assessment, CL’s scores fell below the cut-off in all the categories, irrespective of the hand used to execute the action. There were many errors of coordination (e.g., omission of steps, errors in sequence order, conduit d’approche, and perplexity) and in the spatial orientation of hands and objects (i.e., misplacing of action with respect to the body) and in the positioning of objects (for qualitative analysis of errors in apraxia, see Scandola et al., 2021). He complained about his clumsiness after committing errors in action sequences. A partial recovery was recorded at 4 months, and, in actions executed by the left hand, his performance was above cut-off in meaningful actions involving the maintenance of posture and fingers (e.g., the sign of “victory”; for the distinction between meaningful and meaningless actions, see Canzano et al., 2016; De Renzi et al., 1980).

#### Ideational apraxia

CL presented with severe ideational apraxia at the first assessment. Although he improved by the second assessment, his score still fell below the cutoff for normal performance.

#### Constructional apraxia

Although CL failed in the task involving planning elements, his performance was perfect when he only had to complete a pre-set drawing, confirming deficits in planning but not motor execution.

No signs of *bucco-facial apraxia* were recorded (Table 3).

There was a relevant impact of these deficits on the autonomy of daily life, so that initially CL was not independent in any activities (Table 3, Functional Independence Measure – FIM). Despite the persistence of GS symptoms and only a partial recovery of apraxia, the patient learned compensatory strategies, and, at 4 months, the basic autonomies were largely recovered.

### Neuroanatomical Investigation

**Fig. 1:**
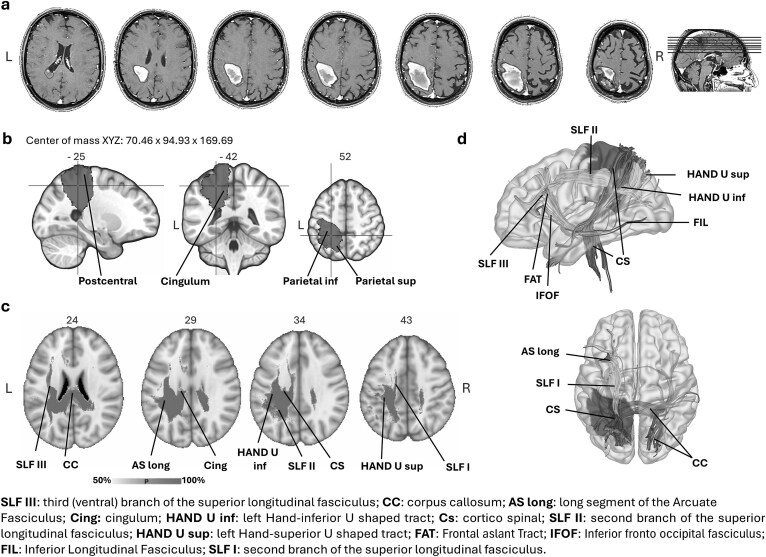
CL’s lesion. (a) The delineation on MRI axial slices. (b) Sagittal, coronal and axial views of the center of mass. (c) White matter indirect disconnections. (d) Medial and dorsal view of the white matter fibers passing through the center of mass. Lesion’s volume in red. L = left, R = right.

Damage was mainly localized in the post-rolandic areas, in particular, in the parietal superior and inferior cortices, and, medially, in the precuneus. More anteriorly, the lesion extended to the peri-rolandic cortices (pre-central and post-central areas) and on the medial side, to the paracentral lobule and cingulum (Fig. 1 and see Supplementary material online, SM 3). Peri-rolandic areas were also disconnected due to the involvement of hand-U tracts. There were no signs of involvement of temporal areas (see Supplementary material online, SM − Table S2).

An indirect involvement of frontal structures was suggested by the disconnection of the long segment of the arcuate fasciculus and the three branches of the superior longitudinal fasciculus (SLF). In particular, SLF I originates in the superior and medial parietal cortex and reaches the dorso-medial frontal regions and the supplementary motor area. SLF II links the inferior parietal lobule (angular gyrus) with ventrolateral and dorsolateral frontal areas. Finally, the most ventral SLF III connects the supramarginal gyrus with the ventral premotor and prefrontal cortex (Petrides & Pandya, 1984; Thiebaut de Schotten et al., 2011). An involvement of the posterior part of the corpus callosum was also found.

## DISCUSSION

The rarity of patients suffering from all four GS symptoms and the repeated observation of CL’s symptoms over time make this single case study of interest in the debate surrounding the syndrome.

An evident symptom was CL’s left–right disorientation relating to his own body and another person’s body. Indeed, he had problems in distinguishing left from right body parts and hands (see Supplementary material online, SM 1). He understood the task, but could not decide which hand to use, and he did not use any alternative strategies (e.g., marks on the skin, watch position) or motor automatisms (e.g., for writing or combing) (Rusconi, 2018). When asked to touch a body part, he was precise in identifying the part (i.e., no autotopoagnosia) but chose the side at random. This left–right confusion was not attributable to a generalized spatial disorder, because he performed the visual–spatial tasks correctly and showed no symptoms of neglect or topographical mistakes in spatial orientation. Neither can a mental rotation deficit explain the symptom that was present in both direct and mirrored conditions.

Finger agnosia was unilateral (right hand) and mainly present when the hand was not visible (Mayer et al., 1999; Mazzoni et al., 1990; Tucha et al., 1997). This was not due to an impairment in interhemispheric transmissions, which would result in an opposite pattern (i.e., agnosia for the left hand). Moreover, severe tactile deficits were excluded by the neurological assessment. As Gerstmann originally reported, CL failed particularly during the identification of the three medial fingers (index, middle, and ring; Gerstmann, 1940; Mayer et al., 1999). Along with these two body-related symptoms, the other two cognitive deficits were recorded (i.e., dysgraphia and dyscalculia).

Cognitive models for calculation (e.g., McCloskey, 1992) distinguish number processing (lexical and/or syntactic errors) and calculation, with errors respectively in the lexical and semantic areas regarding numbers or in arithmetical facts and procedures. Our patient’s disorder mainly affects the latter. Finally, the patient was initially totally unable to write, whereas reading was preserved. Despite a partial improvement on the 4th-month assessment, he still showed difficulties and needed time to think about the shape of the letters. We suspect, he mainly suffered from a peripheral writing deficit involving his graphomotor knowledge of handwriting, with relative sparing of orthographic knowledge (Basagni et al., 2021; Cubelli & Rusconi, 2022).

Along with GS symptoms, CL suffered from limb apraxia involving all typologies of action (meaningful, meaningless actions, actions involving postures or sequences, transitive and intransitive actions, for finger and hand gestures, see Canzano et al., 2016 and De Renzi et al., 1980). In the past, an association between GS and limb apraxia has been suggested (Herrmann & Pötzl, 1926) with the idea that a basic apraxic defect could cause three of the four GS symptoms (finger agnosia, dyscalculia, and dysgraphia). However, limb apraxia in GS has been reported with reference to finger movements (Moro et al., 2009) and constructional apraxia (Gerstmann, 1927; Hayashi et al., 2013; Kinsbourne & Warrington, 1963) rather than to global deficits in gestures (i.e., for all the typologies of hand and finger actions) such as those shown by CL. Motor deficits were really mild and not enough to explain CL’s symptoms. For this reason, we cannot exclude the possibility that limb apraxia is a consequence of GS (and not vice versa, as suggested by Herrmann & Pötzl, 1926), in particular of finger agnosia and left–right disorientation (Buxbaum et al., 2000; Canzano et al., 2016). Alternatively, the two clinical conditions manifested in our patient may be simply an effect of CL’s extensive lesion that underlies both the symptoms due to the proximity and perhaps partial overlap of the networks involved. In particular, the fronto-parietal disconnection associated with the involvement of the three branches of the SLF probably plays a critical role in apraxia (Timpert et al., 2015).

Other pathological scores recorded in the neuropsychological assessment (in the Comb and Razor test and in the Digit span forward) are not confirmed by CL’s performance in other tasks assessing personal neglect and short-term memory (Table 1).

The GS and apraxia symptoms only partially recovered at the 4-month assessment (Tables 2 and 3). Nevertheless, rehabilitation had a critical role in the patient’s recovery of autonomy in daily life activities. The comparison between the FIM scores recorded at admission and discharge showed a significant improvement: in the items related to feeding and use of cutlery the scores improved from 1 to 7 (i.e., total recovery), in activities related to morning hygiene from 1 to 6, in dressing from 1 to 5 and in grooming and personal hygiene from 1to 4. Furthermore, during the execution of the tests for apraxia, the observation of the quality of the patient’s gestures revealed a reduction in the number of errors, especially in terms of conduit d’approche, perplexity, spatial orientation and verbalization.

CL’s damage extended beyond the left inferior parietal lobe typically associated with GS (with only 1.7% of voxels damaged in the angular gyrus), toward the superior parietal cortex and medially to the precuneus and the cingulum. Rostrally, it reached the peri-rolandic cortices. Furthermore, the white matter investigations showed a high probability of disconnections of the angular gyrus and the supramarginal gyrus due to the involvement of SLF II and SLF III, and thus of the fronto-parietal networks. Finally, an involvement of the posterior part of the corpus callosum was found. Taken together, these data support previous results from neuropsychology studies (Basagni et al., 2021; Ranzini et al., 2023) and meta-analytic and combined structural–functional analyses (Rusconi, 2018; Shahab et al., 2022) that consider GS as a disconnection syndrome and not as the consequence of discrete grey matter lesions. Although data on a group of patients would be needed, CL’s neuroanatomical investigation supports the involvement of a core network for GS (Shahab et al., 2022), comprising the left superior parietal lobe (7PC) and the medial and anterior inferior parietal cortices and their white matter connections (intraparietal tracts, fronto-parietal long tracts, Kleinschmidt & Rusconi, 2011). The fronto-parietal disconnections could explain limb apraxia (Canzano et al., 2016).

In his paper, Gerstmann described the syndrome as a body representation disorder, limited to hands and fingers. He proposed that difficulties in finger differentiation and body laterality were strictly connected and could interfere with writing and numerical skills. This interpretation of the syndrome fits very well with CL’s symptoms, which seem in some way to exclude other possibilities. The hypothesis of a disorder in the mental manipulation of information (Gold et al., 1995) or the assumption of damage to the ability to verbally mediate visual and somatosensory spatial knowledge (Ardila, 2014) are excluded by the absence of visuo-spatial and verbal deficits, but also by the lack of differences in CL’s performance in left–right orientation or finger identification for his own or another person’s body. Furthermore, other body representation disorders were excluded by the neuropsychological assessment.

The main limitation of the study is intrinsically related to its nature of single-case study, which is due to the extreme rarity of the syndrome and the difficulty of studying patients in a longitudinal perspective. For this reason, a clinical rather than an experimental approach has been used, in order to emphasize an in-depth analysis of the tetrad of symptoms. Related to this, we acknowledge that another limitation is represented by the fact that, differently from other cognitive domains, auditory working memory has been assessed using just one test (i.e., the Digit span backward test).

## CONCLUSIONS

Overall, the paper offers a framework for the assessment of GS, potentially useful for both clinical and experimental further investigations and for the implementation of group studies in the future. Furthermore, the study suggests that GS symptoms, as well as other body representation disorders (Moro et al., 2023; Pacella et al., 2019), are not transient, thus highlighting the importance of monitoring them over time to understand their impact on patient recovery.

In conclusion, the case described here supports the hypothesis that the four symptoms originally described are interconnected and constitute one syndrome that, as suggested by Gerstmann, has its neural core in a left fronto-parietal network. From a clinical perspective, our data underline the need for a specific, systematic assessment of GS symptoms and their evolution. An integrated approach combining clinical observations, neuropsychological testing and neuroanatomical investigation represents the best strategy to investigate and study clinical conditions that otherwise would remain undiagnosed and misinterpreted.

## Supplementary Material

Supplementary_Material_acaf083
