# Supplementary material for: Gerstmann’s Syndrome and Limb Apraxia: A Single Case Study
Source: Arch Clin Neuropsychol. 2025 Sep 18;40(8):1667–76. doi: 10.1093/arclin/acaf083 (PMC12644058; doi:10.1093/arclin/acaf083)
Supplement: Supplementary_Material_acaf083 [file supplementary_material_acaf083.docx]

**Supplementary Material to**

**Gerstmann’s Syndrome and limb apraxia: a single case study**

**SM 1:** SM 1 - Video 1.mpg - [Supplementary Materials for Gerstmann’s Syndrome and limb apraxia a single case study](https://univr-my.sharepoint.com/:f:/g/personal/maddalena_beccherle_univr_it/EoDXfCvFSGtFjPhCR-w819ABlwuBqNMM5iXXu-MH3kPaQg)

**SM 2:** SM 2 – Video 2.mpg - [Supplementary Materials for Gerstmann’s Syndrome and limb apraxia a single case study](https://univr-my.sharepoint.com/:f:/g/personal/maddalena_beccherle_univr_it/EoDXfCvFSGtFjPhCR-w819ABlwuBqNMM5iXXu-MH3kPaQg)

**SM 3:** Details on the neuroanatomical investigation.

**SM 3.1. CL’s lesion on the MNI152 template.** The lesion was manually drawn on the MNI scan of the patient and then normalised on the MNI152 template.


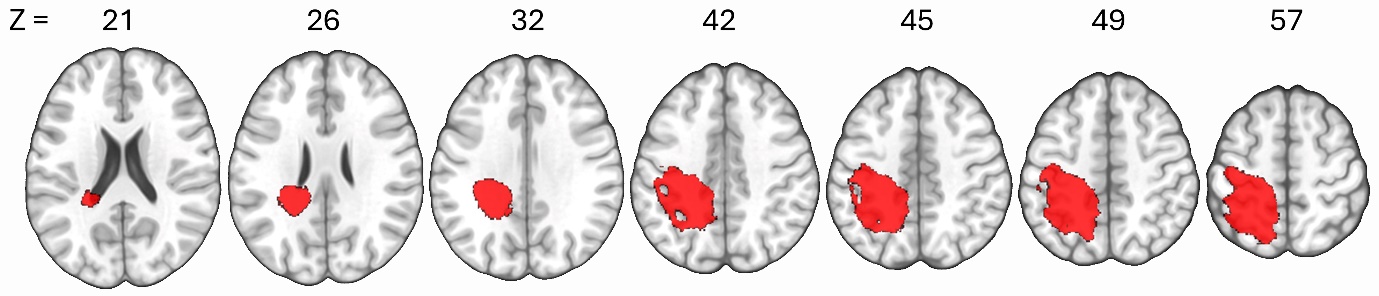


**SM 3.2. CL’s grey matter structures encompassed by the lesion.** Number of voxels (N> 0) and percentage volume (%N > 0) affected by the lesion for each grey matter structure, as reported on MRIcron (AAL atlas) of the patient.

| **Area** | **N voxels > 0** | **N% voxels > 0** |
| --- | --- | --- |
| Precentral_L | 2268 | 8.1 |
| Cingulum_Mid_L | 1923 | 12.4 |
| Cingulum_Post_L | 27 | 0.7 |
| Occipital_Mid_L | 186 | 0.7 |
| Postcentral_L | 11400 | 36.7 |
| Parietal_Sup_L | 10373 | 62.8 |
| Parietal_Inf_L | 8097 | 41.6 |
| Angular_L | 155 | 1.7 |
| Precuneus_L | 9252 | 32.6 |
| Paracentral_Lobule_L | 2190 | 20.2 |

**SM 3.3. CL’s indirect white matter disconnections:** **probability of disconnection.**

The indirect white matter investigation revealed the mean probability of being disconnected for each white matter tract from the atlas (Rojkova et al., 2016). The investigation was performed with a 0.5 threshold using the Disconnectome Maps tool (part of the BCBToolkit software; Foulon et al., 2018).

| **Tracts** | **Mean probability %** |
| --- | --- |
| SLF I L | 77 |
| SLF II L | 80 |
| SLF III L | 77 |
| Cingulum L | 76 |
| Cingulum anterior L | 74 |
| Cingulum posterior L | 62 |
| Uncinate L | 53 |
| Arcuate long segment L | 83 |
| Arcuate anterior segment L | 74 |
| Arcuate posterior segment L | 69 |
| Inferior fronto occipital fasciculus L | 70 |
| Corpus callosum | 75 |
| Cortico-spinal L | 77 |
| Anterior thalamic radiations L | 74 |
| Fronto-striatal L | 72 |
| Fronto pontine L | 76 |
| Paracentral U tract L | 60 |
| HandSup U tract L | 66 |
| Handinf U tract L | 70 |
| Face U tract L | 69 |
| Frontal aslant Tract L | 68 |
| Fronto Insular tract 3 L | 55 |
| Fronto Insular tract 4 L | 71 |
| Fronto Insular tract 5 L | 70 |
| Frontal superior longitudinal L | 70 |
| Frontal inferior longitudinal L | 65 |
| Frontal orbito polar L | 50 |
| Fronto Marginal tract L | 51 |
